# Mathematical Modelling Highlights the Potential for Genetic Manipulation as an Adjuvant to Counter Efflux-Mediated MDR in *Salmonella*

**DOI:** 10.1007/s11538-022-01011-9

**Published:** 2022-04-05

**Authors:** George Youlden, Helen E. McNeil, Jessica M. A. Blair, Sara Jabbari, John R. King

**Affiliations:** 1 School of Mathematics, University of Birmingham, Birmingham, B15 2TT UK; 2 School of Mathematical Sciences, University of Nottingham, Nottingham, NG7 2RD UK; 3 Institute of Microbiology and Infection, University of Birmingham, Birmingham, B15 2TT UK

**Keywords:** *Salmonella*, Efflux pumps, Resistance-nodulation-division, Mathematical modelling, Parameter fitting

## Abstract

Bacteria have developed resistance to antibiotics by various mechanisms, notable amongst these is the use of permeation barriers and the expulsion of antibiotics via efflux pumps. The resistance-nodulation-division (RND) family of efflux pumps is found in Gram-negative bacteria and a major contributor to multidrug resistance (MDR). In particular, *Salmonella* encodes five RND efflux pump systems: AcrAB, AcrAD, AcrEF, MdsAB and MdtAB which have different substrate ranges including many antibiotics. We produce a spatial partial differential equation (PDE) model governing the diffusion and efflux of antibiotic in *Salmonella*, via these RND efflux pumps. Using parameter fitting techniques on experimental data, we are able to establish the behaviour of multiple wild-type and efflux mutant *Salmonella* strains, which enables us to produce efflux profiles for each individual efflux pump system. By combining the model with a gene regulatory network (GRN) model of efflux regulation, we simulate how the bacteria respond to their environment. Finally, performing a parameter sensitivity analysis, we look into various different targets to inhibit the efflux pumps. The model provides an *in silico* framework with which to test these potential adjuvants to counter MDR.

## Introduction

### Efflux Mediated Antibiotic Resistance

Antibiotics are natural or synthetic drugs that are used to treat bacterial infections by killing bacteria or preventing them from growing (Walsh et al. 2003). The widespread use of antibiotics, however, has resulted in the selection for antibiotic resistant strains of bacteria that are problematic to treat (Davies and Davies 2010). Bacteria develop resistance by adapting in the presence of antibiotics; factors that determine this include the dosage and how frequent an antibiotic is administered. In 2015 the World Health Organisation (WHO) stated that without immediate global action, the world is headed towards a post-antibiotic era (World Health Organization 2015).

Bacteria have developed resistance to antibiotics by various mechanisms, notable however, is the use of permeation barriers and the expulsion of antibiotics via efflux pumps (Nikaido 1998). Efflux was first identified as a resistance mechanism for tetracycline in *Escherichia coli (E. coli)* (McMurry et al. 1980). There are six types of bacterial efflux pumps that are capable of expelling antibiotics, these are the major facilitator (MF) superfamily, the adenosine triphosphate (ATP) binding cassette (ABC) family, the small multidrug resistance (SMR) family, the multidrug and toxic compound extrusion (MATE) family, the proteobacterial antimicrobial compound efflux (PACE) transporter family and finally the resistance-nodulation-division (RND) family (Alav et al. 2021). The RND family can be found in Gram-negative bacteria, they facilitate expulsion of a wide range of substrates including antibiotics which contributes to multidrug resistance (MDR) (Nikaido 1998). Due to the existence of RND efflux pumps and a dual membrane structure, Gram-negative bacteria intrinsically less sensitive to antimicrobials than Gram-positive bacteria (Li et al. 2016). Over-expression of RND efflux pumps commonly confers MDR in bacteria, an example of this is the overexpression of the AcrAB-TolC efflux pump in *E. coli* and *Salmonella* (Blair et al. 2014). This over-expression is often controlled by mutations within gene regulatory networks (GRNs) that govern the expression of efflux pump proteins (Webber and Piddock 2003).

### RND Efflux Pumps in *Salmonella*

*Salmonella* spp. is a genus of rod-shaped Gram-negative bacteria that is comprised of two species *Salmonella*: *S. enterica* and *S. bongori* (Tindall et al. 2005). These species exist worldwide and are responsible for causing gastroenteritis, septicaemia and enteric fever (Zhang et al. 2003), for example. Estimates show that *Salmonella* gastroenteritis is annually responsible for 93.8 million illnesses and 155,000 deaths worldwide (Majowicz et al. 2010). *Salmonella enterica* serovar Typhimurium (S. Typhimurium) is one of the leading causes of non-typhoidal salmonellosis. MDR S. Typhimurium strains have been shown to over-express proteins forming the RND efflux pump system AcrAB-TolC, exhibiting resistance to fluoroquinolone, chloramphenicol–florfenicol and tetracyclines (Baucheron et al. 2002, 2004).

**Fig. 1:**
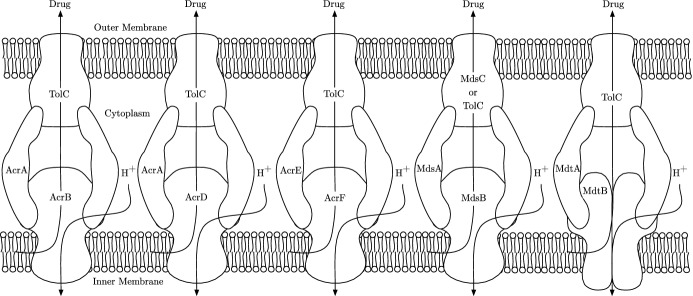
The five RND efflux pump systems found in *Salmonella*. Each of these pumps export drugs from within the cell via proton motive force, driven by the electrochemical gradient caused by hydrogen ions $(H^{+})$ moving into the cytoplasm (Piddock 2006)

From genomic analysis, it has been shown that *Salmonella* strains contain five RND efflux pump systems, AcrAB, AcrAD, AcrEF, MdsAB and MdtAB (Horiyama et al. 2010) (Fig. 1). Various antibiotics have been shown to be substrates of multiple of these efflux pump systems (Nishino 2016). A potential novel treatment strategy to combat resistance is through the use of efflux pump inhibitors (EPIs) (Martínez 2012). EPIs inhibit the action of efflux pumps; they can target efflux pumps directly (efflux inhibitors), or can be used indirectly to target gene expression (efflux modulators) (Gill et al. 2015). Their main purpose is to increase the intracellular concentration of antibiotic, such that the bacteria are unable to survive (Sharma et al. 2019). The development of novel EPIs is considered a promising strategy to make a bacterium more sensitive to antibiotics and reverse MDR in Gram-negative pathogens (Spengler et al. 2017; Blanco et al. 2018). However in *Salmonella*, it has been demonstrated that inhibition of one of the RND efflux pump systems results in drug expulsion through another RND efflux pump system, commonly as a result of gene over-expression in the alternative system (Nishino et al. 2006; Hirakawa et al. 2008; Blair et al. 2014). Therefore, effective inhibition of efflux may involve multiple targets. A theoretical model of these interacting systems will accelerate our understanding of how best to approach this.

### Mathematical Models of Cellular Diffusion and Efflux

In order to model efflux, we must consider the diffusion of the antibiotic between the intracellular and extracellular spaces. Cellular drug diffusion models have primarily been used to model the drug diffusion in tumour cells. For an overview of some of these models, see Kim et al. (2013). More closely related to this study however, Rahman et al. (2018) present a simple model of a spherical tumour cell with a depleting outer boundary condition. Here, they model the case where a drug is injected to the centre of the cell and diffuses outwards. They are then able to produce links between the intracellular concentration to cell death. Yi et al. (1999) develop a single cell model to look into the diffusion and efflux of MDR cancer cells. This model also includes a supply of drug via injection. Efflux is modelled through active transport using Michaelis–Menten equations. Michelson and Slate (1992, 1994) present models of the p-glycoprotein pump, associated with MDR in cancer patients. This pump is energy dependent relying on dephosphorylation processes to function. Notably, this efflux pump functions differently to the proton motive force driving the RND efflux pumps in this study.

In terms of spherical drug diffusion models, many models have been produced to investigate the dynamics of drug delivery capsules. Wang et al. (2010) produce a model of diffusion in a spherical drug delivery device, surrounded by a finite medium. This finite medium is defined by a no flux outer boundary, i.e. there is no drug depletion. The model is effectively fitted to data to achieve drug diffusion coefficients. Kaoui et al. (2018) produce a similar model governing the release of drug from a multi-layer spherical capsule as a drug delivery system. This capsule is surrounded by an outer shell that protects the release of drug into the outside medium. A PDE model is produced, with boundary conditions imposed to include the dynamics of diffusion between the various layers and the outer shell. A two-layer capsule is first modelled, with the extension of the solution procedure shown to produce an infinite number of layers.

With regard to related efflux models, Perez et al. (2017) develop an ODE model of efflux in *E. coli* by TetB. This type of efflux pump spans one membrane. Their model has diffusion through two membranes of the antibiotic into the cytoplasm, with efflux from the cytoplasm to the periplasm. Diao et al. (2016) produce an ODE model of a yeast efflux pump found in *Saccharomyces cerevisiae*. They model the negative feedback loop of a regulator, efflux pump and inducer (a substrate of the efflux pump). Charlebois et al. (2014) also produce an ODE model of the efflux pump in *Saccharomyces cerevisiae*. Here, a more complex model is produced, consisting of three genes that are part of a drug resistance network involved with efflux pump expression.

Concerning models of the five RND efflux pumps found in *Salmonella*, these mainly revolve around the dominant pump AcrAB which is also found in *E. coli*. Nagano and Nikaido (2009) exhibit an ODE model of antibiotic efflux via AcrAB-TolC in *E. coli*. Here, they model diffusion via Fick’s Law and model transport via Michaelis–Menten kinetics. Parameter fitting is used to obtain binding coefficients for various antibiotics. Lim and Nikaido (2010) extend this work further to find binding coefficients for penicillins. Rossi et al. (2018) produced a model of the regulation of MarA, the main activator of *acrAB* expression in *E. coli*. Analysis from this model showed the resulting effects on downstream genes including *acrAB*. Langevin and Dunlop (2018) exhibit a model showing the stress tolerance involved in expressing AcrAB in *E. coli*. In this model, they compared wild-type and *acrAB* knockout strains. They varied the levels of environmental stress and measured the resulting effects on population size.

Finally, Youlden et al. (2021) produced a model of the GRN governing the expression of the efflux pumps AcrAB and AcrEF in *Salmonella*. A time-dependent asymptotic analysis was performed, breaking down the ODE model into a step by step model. By performing this analysis, they were able to identify the most suitable targets within the network for combating antibiotic resistance as well as information about when inhibition methods should be deployed. However, this model did not explicitly consider the concentration of antibiotic and how this impacts the GRN. We address this in the current study by developing a substrate efflux model that we then link with the existing (but updated) GRN model. For this, we consider a cellular model of substrate efflux governed by the RND family in *Salmonella*. This cellular model is derived using a similar approach to the spherical capsule diffusion models previously mentioned. Using experimental data, we first parametrise this model using a simplified equation governing the regulation of the efflux pump systems, later replacing this equation with the more complex GRN model. We are then able to conduct analysis into how the cells are able to react to their environments, enabling us to identify potential targets to counter efflux-mediated MDR.

## Model Formulation

### Experimental Protocol

We base our mathematical model upon experiments completed by the Blair laboratory, based at the University of Birmingham (McNeil et al. 2019; Smith and Blair 2013). Cultures of *Salmonella* are adjusted to an optical density (measured at a wavelength of 600*nm*, OD600) of 0.2 and are placed into standard 96 well plates. Individual cultures are loaded with high concentrations of ethidium bromide (a substrate of multiple RND efflux pumps). At this stage, an efflux inhibitor (CCCP) is present which dissipates the proton motive force of the RND efflux pumps, resulting in substrate accumulation. Ethidium bromide is a DNA-intercalating agent that fluoresces when it is bound to DNA (Duhamel et al. 1996) and is commonly used as a measurable substitute for antibiotics when investigating efflux systems. Ethidium bromide dye has been used by many research teams to measure the activity of efflux through RND pumps. Examples include (Blair and Piddock 2016; Paixão et al. 2009; Pal et al. 2020; Viveiros et al. 2008; Ma et al. 1995). By measuring this fluorescence (measured in arbitrary units), the concentration of ethidium bromide within a culture can be approximated. The cells are washed to remove extracellular substrate and are then re-energised with glucose so that the efflux pumps begin to extrude the substrate. The fluorescence is then measured over  1 hour (Blair and Piddock 2016). Experiments were performed on *Salmonella* strains, including those lacking various efflux genes to see the differences caused by expressing various efflux pump systems. For these experiments, four RND efflux pumps were considered: AcrAB, AcrEF, MdsAB and MdtAB. All of these efflux pump proteins share the same outer membrane protein TolC, with MdtAB also having the ability to form a system with the outer membrane protein MdtC (Nishino 2016).

### Cell Efflux Model

To formulate our model, we assume that each cell in a population acts identically, i.e. we consider a single cell that represents an ‘average’ of the population. We also assume that the concentration of ethidium bromide is evenly distributed in the population such that every cell has an identical initial concentration of ethidium bromide. To reduce model complexity, we assume this distributed concentration of ethidium bromide is localised and independent to each cell, meaning that if the substrate has been expelled into the extracellular space, it can diffuse back into the original cell. We consider ethidium bromide in two states. The first state is ethidium bromide that fluoresces because it is bound to DNA, we will further refer to this state as bound ethidium bromide. In this state the ethidium bromide cannot pass through the cell membrane via efflux or diffusion. The second state is ethidium bromide that is not bound to DNA. We will further refer to this state as unbound ethidium bromide. In this state, ethidium bromide does not fluoresce and can move freely through the cell membrane via efflux or diffusion. Since one state is bound, we assume that the two states will diffuse at different rates and hence apply a different diffusion coefficient for each state. We assume a directly proportional relation between the fluorescence of ethidium bromide and the concentration of bound ethidium bromide.

**Fig. 2:**
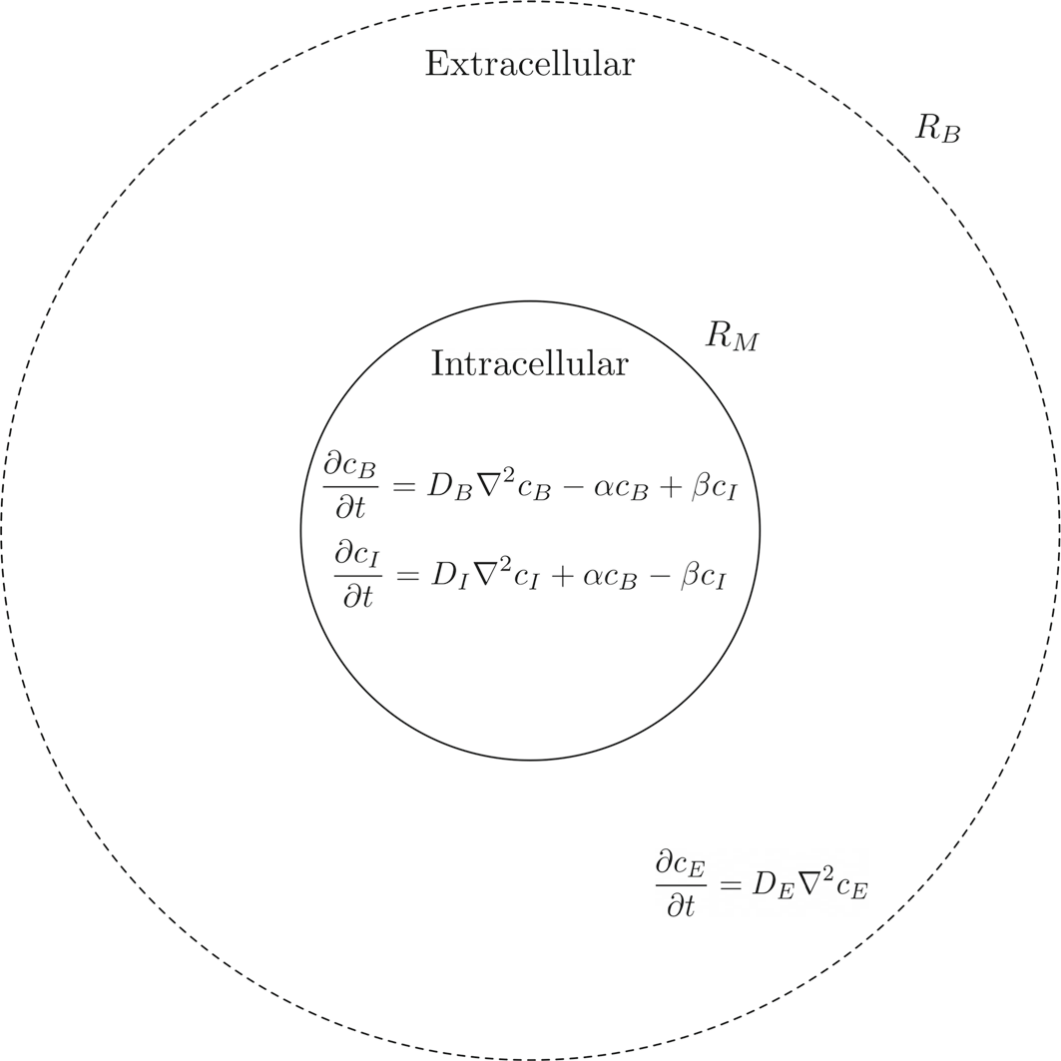
A schematic of the cell efflux model. The solid line represents our cell membrane at radius $R_M$, whereas the dashed line represents our outer boundary at radius $R_B$. We have placed our equations where they apply in the intracellular and extracellular space

We exhibit a schematic of our model in Fig. 2. We consider the cells to be spherical and axisymmetric and hence use spherical coordinates with intracellular space bounded at radius $R_M$. This is surrounded by extracellular space with an outer boundary of radius $R_B$. At the cell radius, we have a thin permeable membrane which contains all of our efflux pump systems that expel unbound substrate from the intracellular to the extracellular space. Whilst *Salmonella* are rod-shaped bacteria, we opt to model them spherically for simplicity in implementing boundary conditions, but note that the model is generalisable to alternative coordinate systems. The equations for the model are a system of partial differential equations (PDEs) as follows

1 $$\begin{aligned} \frac{\partial c_B}{\partial t}&=D_B \nabla ^2 c_B-\alpha c_B+\beta c_I, \end{aligned}$$

2 $$\begin{aligned} \frac{\partial c_I}{\partial t}&=D_I \nabla ^2 c_I+\alpha c_B-\beta c_I, \end{aligned}$$

3 $$\begin{aligned} \frac{\partial c_E}{\partial t}&=D_E \nabla ^2 c_E. \end{aligned}$$

Here, $c_B$ denotes the bound concentration of substrate, $c_I$ denotes the intracellular unbound concentration of substrate, and $c_E$ denotes the extracellular unbound concentration of substrate. Each concentration diffuses at an independent rate $D_B, D_I$ or $D_E$. In addition, the intracellular ethidium bromide undergoes DNA binding at rate $\beta$ and unbinding at rate $\alpha$. We assume that there is always a sufficient concentration of DNA that is well mixed throughout the cell such that ethidium bromide is able to bind at all times.

We note that under these assumptions, the model can also be formulated as a compartmentalised ODE system. By formulating and comparing simulations of the two models, we have been able to show that the ODE model works and behaves similarly (but not identically) to the PDE model (see Youlden 2021 for details). We choose, however, to maintain the more mechanistic PDE model as we are able to visualise and analyse the profile of the drug. In addition, it is also much easier to generalise the model to other spatial dependencies and include additional flows, such as those around a wound or infection site, that may be present outside of a laboratory experiment setting. Furthermore, the use of the PDE model in its current form could be important in future investigations into potential therapies or substrates with different diffusivities for which the ODE model may fail to encapsulate the correct behaviour, for example.

#### Boundary Conditions

The boundary conditions at $r=0$ are:

4 $$\begin{aligned} \left. D_B\frac{\partial c_B}{\partial r}\right| _{r=0}=0, \quad \left. D_I\frac{\partial c_I}{\partial r}\right| _{r=0}=0. \end{aligned}$$

Here, we assume axisymmetric properties of the cell for the intracellular concentrations. This also avoids singularities about the point $r=0$. We note that the substrate cannot cross the membrane when it is bound; hence, we set a no flux boundary for $c_B$ here:

5 $$\begin{aligned} \left. D_B\frac{\partial c_B}{\partial r}\right| _{r=R_M}=0. \end{aligned}$$

**Fig. 3:**
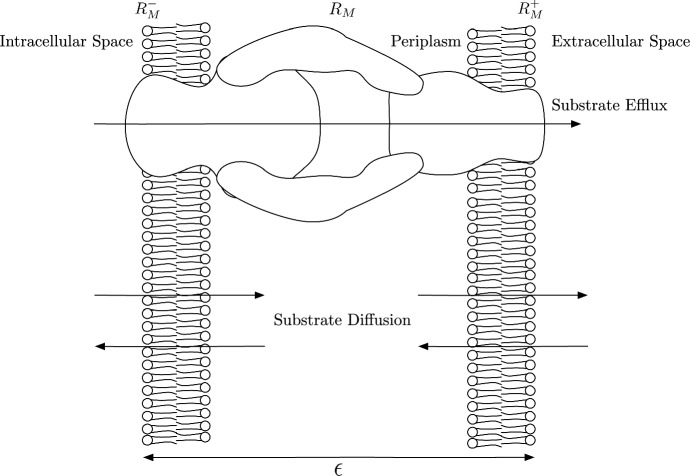
A schematic showing the processes involved at the membrane $R_M$ with small finite thickness $\epsilon$. We show fictitious points $R_M^-$ and $R_M^+$ that are part of the intracellular and extracellular space, respectively. Efflux of substrate is directly from the intracellular space to the extracellular space through the RND efflux pumps. Diffusion of substrate is in both directions through each membrane from the intracellular and extracellular spaces into the periplasm

$c_I$ and $c_E$, however, are free to cross the membrane. We demonstrate the properties of the membrane in Fig. 3. Here, the membrane has small but finite thickness $\delta$, with the fictitious points $R_M^-$ and $R_M^+$ being the intracellular and extracellular points of the membrane, respectively. We can see that we have both diffusion and efflux through the membrane and thus the total flux through the membrane will be a combination of diffusive and advective flux through efflux. Via Fick’s law (1855), we can calculate the diffusive flux through the membrane:

6 $$\begin{aligned} J_D&=-D_M \nabla c(R_M,T), \\&=-D_M \frac{c(R_M^+,T)-c(R_M^-,T)}{\epsilon }, \nonumber \\&=\frac{D_M}{\epsilon } (c_I(R_M,T)-c_E(R_M,T)).\nonumber \end{aligned}$$

Here, $D_M$ denotes the diffusion coefficient of substrate inside the membrane. In regards to the advective flux, this is proportional to the product of substrate concentration and the volume flow (Koch 1970)

7 $$\begin{aligned} J_A&=X \, c(R_M^-,T),\\&=X \, c_I(R_M,T).\nonumber \end{aligned}$$

Here, *X* represents the volume flow caused by active transport via the efflux pump systems. Taking the combination of fluxes, the total flux through the membrane is

8 $$\begin{aligned} J&=J_D+J_A, \\&=P \, (c_I(R_M,T)-c_E(R_M,T))+X \, c_I(R_M,T).\nonumber \end{aligned}$$

Here, $P=\dfrac{D_M}{\epsilon }$ is a mass transfer coefficient related to the permeability of the membrane. We require continuity of flux at the membrane and therefore the boundary condition at $R=R_M$ can be written as

9 $$\begin{aligned} -\left. D_I\frac{\partial c_I}{\partial r}\right| _{r=R_M}=-\left. D_E\frac{\partial c_E}{\partial r}\right| _{r=R_M}=(P+X)\,c_I(R_M,t)-P\,c_E(R_M,t). \end{aligned}$$

This condition encompasses flux continuity between the intracellular and extracellular space, with interface conditions that characterise the properties of the membrane which we assume are constant for the full duration of our simulations (Cussler and Cussler 2009). If we set $X=0$ such that there is no efflux, we can see that as $P \rightarrow 0$ this condition becomes a no flux boundary condition (which is expected with no membrane permeability), in contrast as $P \rightarrow \infty$ we approach $c_I(R_M, t)=c_E(R_M, t)$ which models the case with no membrane. In order to implement a feedback mechanism from the bacteria upon the intracellular substrate concentration, we assume that the volume flow *X* is dependent on the antibiotic concentration within the cell which we model as

10 $$\begin{aligned} \frac{\mathrm{d} X}{\mathrm{d}t}&=\phi \, I-\delta X,&X(0)&=X_0,&I(t)&=\frac{3}{R_M^3}\int _{0}^{R_M} r^2 \,c_B(r,t) \,\mathrm{d}r, \end{aligned}$$

where $\phi$ and $\delta$ are the efflux pump formation (or upregulation) rate and degradation (or downregulation) rate, respectively, and *I* is the averaged intracellular concentration of substrate. Note that *X*(*t*) will differ depending on which efflux pumps are active in a given strain—more details are given in Sect. 3.

Finally, we have the outer boundary condition. We assume that the extracellular space is limited, such that substrate expelled out of the cell will always be within range to diffuse back in, as shown by the experimental data. Therefore, we set the outer boundary to be a no flux condition,

11 $$\begin{aligned} \left. D_E\frac{\partial c_E}{\partial r}\right| _{r=R_B}=0. \end{aligned}$$

#### Initial Conditions

As the cultures are washed to remove extracellular substrate before the initial fluorescence is measured, we assume that there is no substrate in the extracellular space initially. In regards to the intracellular space, we do not know the ratio of unbound to bound substrate, thus we introduce the parameter $\gamma$ into our initial conditions that will define the ratio of unbound to bound substrate. The initial conditions for each concentration are as follows

12 $$\begin{aligned} c_B(r,0)=C_{B0}, \quad c_I(r,0) =\gamma \, C_{B0}, \quad c_E(r,0) =0, \end{aligned}$$

where $C_{B0}$ is the initial concentration of bound substrate.

## Parametrisation of the Cell Efflux Model

**Table 1:** A summary of the strains involved in the experiments

Strain name	Active efflux pumps	Variable	Parameters
Wild-type	AcrAB, AcrEF, MdsAB, MdtAB	$X_{AEST}$	$\phi _{AEST}$, $\delta _{AEST}$
A Knockout	AcrEF, MdsAB, MdtAB	$X_{EST}$	$\phi _{EST}$, $\delta _{EST}$
E Knockout	AcrAB, MdsAB, MdtAB	$X_{AST}$	$\phi _{AST}$, $\delta _{AST}$
S Knockout	AcrAB, AcrEF, MdtAB	$X_{AET}$	$\phi _{AET}$, $\delta _{AET}$
T Knockout	AcrAB, AcrEF, MdsAB	$X_{AES}$	$\phi _{AES}$, $\delta _{AES}$
AE Knockout	MdsAB, MdtAB	$X_{ST}$	$\phi _{ST}$, $\delta _{ST}$
AEST Knockout	N/A	N/A	N/A

We list each strain’s active efflux pumps as well as their corresponding efflux variable and parameters. Notably, the ‘T knockout’ strain has inactive MdtAB periplasmic adaptor protein rather than inactive gene *mdtAB*

### Parametrisation Data

Experiments were carried out on different cultures of *Salmonella* with various efflux pump knockouts as described in Sect. 2.1. We summarise all of these strains with their active efflux pumps and their corresponding efflux variable and parameters in Table 1. In regards to efflux variable and parameter notation, we list the efflux pumps that are still active in the subscript (e.g. *A* (or AcrAB) knockout is noted by $X_{EST}$). We include each strain’s efflux parameter in the following parameter fitting exercises by modifying the membrane boundary condition (9) and efflux flow equation (10) for each strain. This enables us to find the corresponding efflux profile for each strain through multiple parameter fitting exercises.

For each experiment with a given strain, we have multiple assays (biological repeats). Within these individual assays, we have technical repeats for each strain. The mean and standard deviations are found of these technical repeats. Data were taken from the timepoint at which fluorescence peaked before reducing as a result of efflux. This ensures that the systems have all stabilised following the glucose injection that re-enables efflux and facilitates better comparison across the assays. In order to combine the data from all assays, we normalise our data by dividing the fluorescence over time by the peak initial fluorescence. As not all assays run for the same length of time (until the data show evidence that the efflux is levelling off), we split our combinations into three time periods (short, medium and long-time). Whilst the short-time plots include data of all assays, the medium- and long-time plots are restricted to the assays that reach the final time point on the corresponding plot.

### Parametrisation Methods

We employ the function *fminsearch* in MATLAB to obtain our parameter estimates. The function uses the Nelder–Mead simplex method (described in Lagarias et al. 1998) to minimise a given objective function starting from initial parameter guesses. In order to obtain these parameter guesses, we use a Latin hypercube method of sampling as described in McKay et al. (1979). On establishing realistic upper and lower parameter bounds, we create a parameter space from which a range of initial parameter guesses are chosen. We use this sampling method in order to choose a well-spread distribution of initial parameter guesses (something that is not guaranteed from using a random method of sampling). The objective function that we attempt to minimise is as follows:

13 $$\begin{aligned} f=\frac{1}{t_\mathrm{max}}\sum \mid y(t)-I(t)) \mid \end{aligned}$$

where

$$\begin{aligned} I(t)&=\frac{1}{\frac{4}{3}\pi R_M^3}\iiint _V r^2 \, \sin (\theta )\, c_B(r,t) \, \mathrm{d}r\,\mathrm{d}\theta \,\mathrm{d}\phi \\&=\frac{3}{R_M^3}\int _{0}^{R_M} r^2 \,c_B(r,t) \,\mathrm{d}r. \end{aligned}$$

Here, *y* denotes the experimental data, *I* denotes the averaged intracellular concentration of bound substrate, and $t_\mathrm{max}$ is the maximum time for the simulations. We divide the objective function by the amount of data points in the corresponding assay to which we are fitting. This gives us a comparison point between the accuracy of fits to different strains that differ in assay length. In addition, we have applied a constraint to the parameters to be nonnegative by ensuring that the absolute value of each parameter is taken through every iteration of finding the objective function. We note that the data values are measured in relative fluorescence and our model in concentration. However, as we assume a directly proportional relationship between the fluorescence and intracellular concentration of substrate, we directly fit the model to the data. Finally, we list all parameters and their units used in this model in Table 2.

**Table 2:** All parameters used in our bound ethidium bromide model and their respective units

Parameter	Description	Value	Units
$R_M$	Membrane radius	2	$\upmu$m
$R_B$	Outer boundary radius	Not set	$\upmu$m
*P*	Permeability mass transfer coefficient	Not set	$\upmu$m min$^{-1}$
$X_{x 0}$	Initial efflux volume flow coefficient for strain with pumps ‘*x*’ Active	Not set	$\upmu$m min$^{-1}$
$\phi _{x}$	Efflux pump formation rate for strain with pumps ‘*x*’ Active	Not set	$\upmu$m min$^{-2}$
$\delta _{x}$	Efflux pump degradation rate for strain with pumps ‘*x*’ Active	Not set	min$^{-1}$
$D_B$	Diffusion coefficient of bound substrate	0	$\upmu$ m$^2$ min$^{-1}$
$D_I$	Diffusion coefficient of unbound intracellular substrate	Not set	$\upmu$m$^2$ min$^{-1}$
$D_E$	Diffusion coefficient of unbound extracellular substrate	Not set	$\upmu$m$^2$ min$^{-1}$
$\alpha$	Unbinding rate of bound substrate	Not set	min$^{-1}$
$\beta$	Binding rate of unbound substrate	Not set	min$^{-1}$
$\gamma$	Initial ratio of unbound to bound substrate concentrations	Not set	N/A

If we are estimating a parameter we will list it as ‘Not Set’ in the ‘Value’ column, otherwise if the parameter is fixed we list its value

In order to produce numerical simulations of the PDE model, we use the forward time centralised space (FTCS) finite difference method (LeVeque 2007; Versypt and Braatz 2014). We set the cell to have a radius of 2 $\upmu$m so that it yields a similar spherical volume and surface area to the typical rod-shaped dimensions of *Salmonella*. We have also set the outer boundary to be at 4 $\upmu$m; this distance has been chosen as it provided the best results in early iterations of the parameter fitting exercises (omitted here for brevity). For the following results, we have fitted to the long-time data only. We withhold the short-time and medium-time assays (where available) for model validation by testing the accuracy of the fit. For simplicity of our parameter fitting exercises, we set our bound ethidium bromide diffusion rate to be zero ($D_B=0$) reducing the number of parameters in the following fits. This is under the assumption that the bound molecules will be large compared to unbound molecules such that the diffusion will be negligible compared to the unbound state. For a complete overview of the numerical method and parameter fitting exercises used to obtain these results, see Youlden (2021).

#### Wild-Type and AEST Knockout

**Fig. 4:**
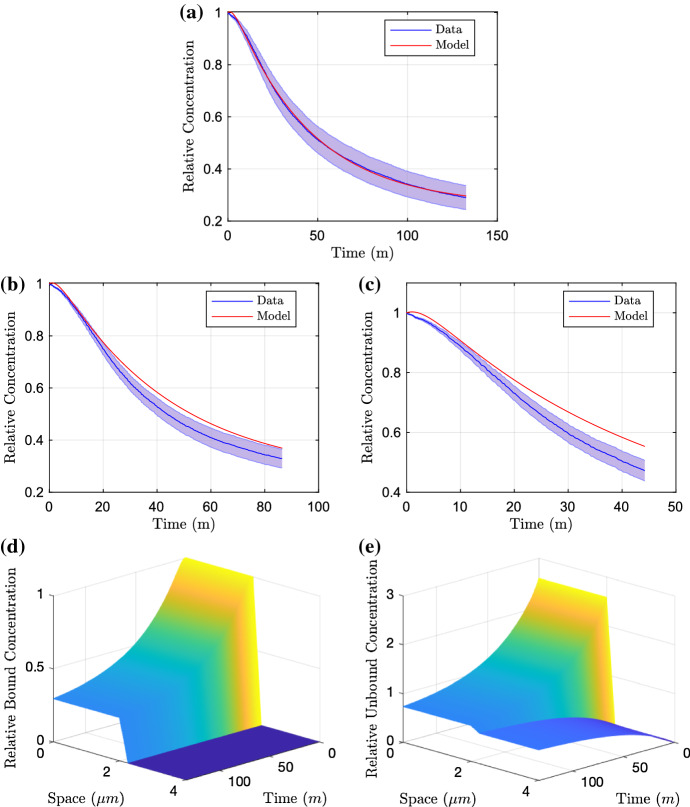
Parameter fitting results of the PDE model fitting to the long-time AEST data (**a**). We test the model against the data for the medium-time and short-time assays in (**b**) and (**c**), respectively. Finally in **d** and **e**, we demonstrate the distribution profiles for both bound and unbound concentrations upon fitting to the long-time data (Color figure online)

**Fig. 5:**
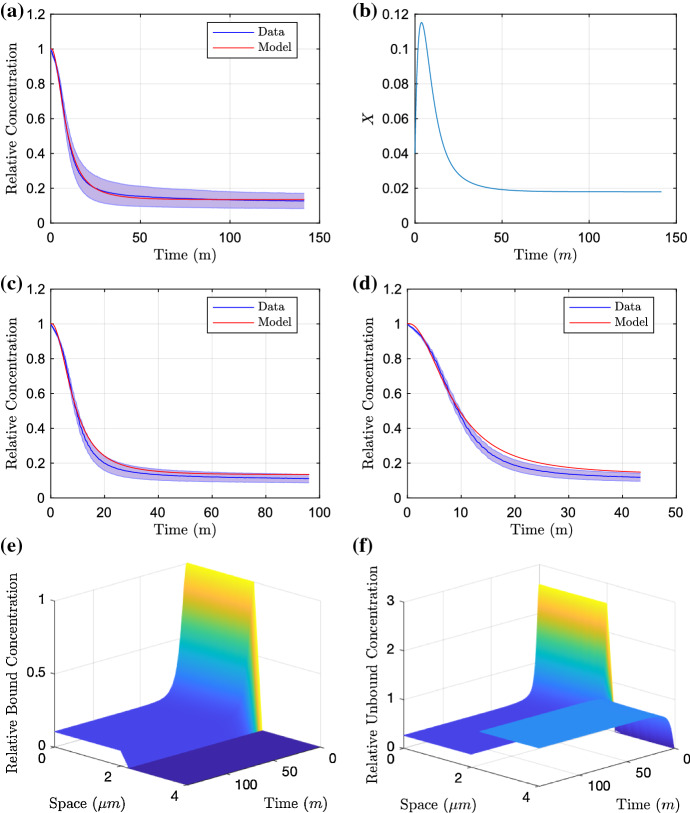
Parameter fitting results of the PDE model fitting to the long-time wild-type data (**a**) with resulting efflux profile (**b**). We test the model against the data for the medium-time and short-time assays in (**c**) and (**d**), respectively. Finally in **e** and **f**, we demonstrate the distribution profiles for both bound and unbound concentrations upon fitting to the long-time data (Color figure online)

We initially fit the model to the data where no RND efflux pumps are active in the strain. Whilst there is the possibility of less significant efflux pump systems being active in this case, efflux contributed from these pumps is likely to be negligible compared to the efflux caused by the RND efflux pumps. Thus by initially fitting to these data, we can construct a base for the diffusion parameters and membrane permeability as these should not vary between experiments. The parameters identified for the AEST are as follows

14 $$\begin{aligned} {[}R_B, D_I, D_E, P, \alpha , \beta , \gamma ]=[4, 2.30, 1.01, 1.47 \times 10^{-2}, 0.22, 8.89 \times 10^{-2}, 2.59],\nonumber \\ \end{aligned}$$

given to three significant figures and resulting in an objective function of $2.44 \times 10^{-4}$. We plot the resulting parameter fit in Fig. 4. We can see that these parameters produce a good fit to the long-time data (Fig. 4a) and whilst the model does not lie within the standard deviation of the medium- and short-time data (Fig. 4b, c), we can see that the model is able to reproduce very similar dynamics. By fixing our optimal parameters from the AEST knockout case, we continue to find the remaining efflux parameters from the other strains. Fitting to the wild-type data, we achieve the parameters

15 $$\begin{aligned} {[}X_0, \phi , \delta ]=[4.00 \times 10^{-2}, 7.99 \times 10^{-2}, 0.60], \end{aligned}$$

given to three significant figures and resulting in an objective function of $3.76 \times 10^{-4}$. We plot the resulting parameter fit in Fig. 5. With the addition of efflux in this case, we can see that again the model provides a good fit to the long-term data (Fig. 5a). In addition to this, good fits are shown against the medium- and short-time data (Fig. 5c, d). The efflux profile is also exhibited in 5b, we see that the initial condition of efflux is small; however, there is a sharp increase in efflux before decreasing to reach steady state.

**Fig. 6:**
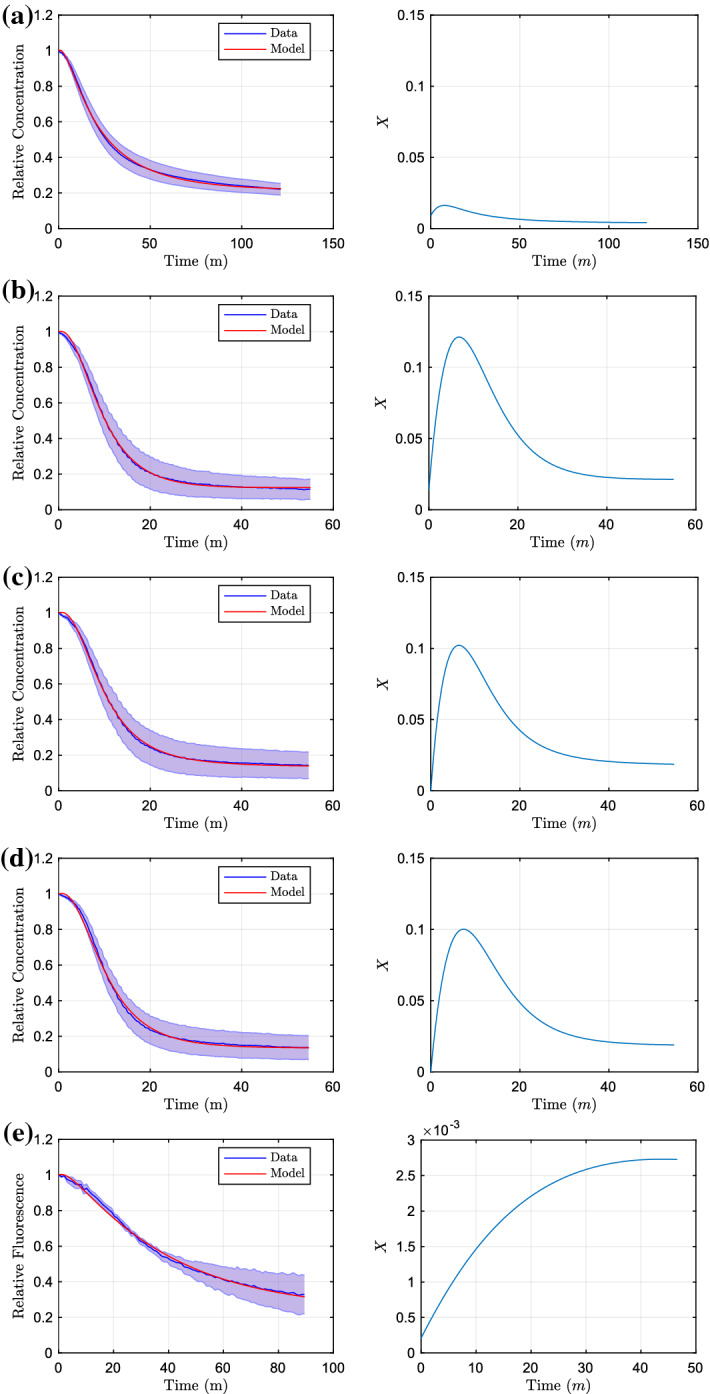
Parameter fitting results of the bound ethidium bromide model to the data of efflux knockouts, with fixed AEST parameters (14). We show the model fits with their resulting efflux knockouts. We show in **a** A knockout, **b** E knockout, **c** S knockout, **d** T knockout and finally, **e** AE knockout. Plots here are only shown on one timescale, due to no experiments taken for shorter or longer time periods (Color figure online)

**Fig. 7:**
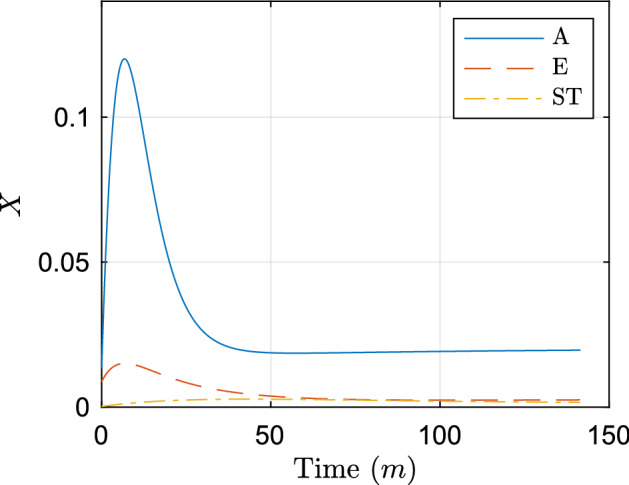
Individual efflux profiles estimated from parameter fitting results. Here, A denotes AcrAB, E denotes AcrEF, and ST denotes MdsAB and MdtAB (Color figure online)

#### Efflux Knockouts

The model is able to encapsulate the behaviour of the two most extreme cases of full and no efflux (wild-type and AEST knockout). We now fit new efflux parameters for each case of efflux knockouts, keeping the AEST parameters fixed (14). For notation, we list all fitted parameters as $X_{G0}$, $\phi _G$ and $\delta _G$ together with their corresponding objective function $\theta _G$, where *G* represents the efflux pumps that are active in that case. The parameters we achieve are

16 $$\begin{aligned} {[}X_{EST0}, \phi _{EST}, \delta _{EST}, \theta _{EST}]= & {} [8.99\times 10^{-3}, 4.89\times 10^{-3}, 0.26, 4.53\times 10^{-4}], \end{aligned}$$

17 $$\begin{aligned} {[}X_{AST0}, \phi _{AST}, \delta _{AST}, \theta _{AST}]= & {} [1.35\times 10^{-2}, 4.18\times 10^{-2}, 0.24, 6.15\times 10^{-4}], \end{aligned}$$

18 $$\begin{aligned} {[}X_{AET0}, \phi _{AET}, \delta _{AET}, \theta _{AET}]= & {} [7.01 \times 10^{-8}, 4.30 \times 10^{-2}, 0.32, 6.53 \times 10^{-4}], \end{aligned}$$

19 $$\begin{aligned} {[}X_{AES0}, \phi _{AES}, \delta _{AES}, \theta _{AES}]= & {} [7.87\times 10^{-8}, 3.42\times 10^{-2}, 0.25, 9.53\times 10^{-4}], \end{aligned}$$

20 $$\begin{aligned} {[}X_{ST0}, \phi _{ST}, \delta _{ST}, \theta _{ST}]= & {} [2.12\times 10^{-4}, 1.57\times 10^{-4}, 2.94\times 10^{-2}, 1.30\times 10^{-3}],\nonumber \\ \end{aligned}$$

given to three significant figures and resulting in a combined objective function of $3.98\times 10^{-3}$. We plot the resulting fits in Fig. 6. We can immediately see that for each efflux knockout case, the dynamics are closely matched with the majority of the model fitting within the standard deviation of the data. It is also interesting to note that nearly all efflux profiles (barring the AE knockout in Figure 6e) starts off at a low value, then rapidly increases before decreasing to a steady-state value. The efflux profile for the AE knockout (Fig. 6e) has a very different efflux profile to the other cases. In this case, only the efflux pumps MdsAB and MdtAB are active, these are both weaker efflux pumps compared to AcrAB and AcrEF, explaining the lower efflux and initial peak. Notably these strains parameters take very different values to the other knockout strains. All other knockout strains containing similar upregulation and downregulation rates $\phi$ and $\delta$, with only the AcrAB knockout having a reduced upregulation rate. This is expected as the efflux pump systems will be regulated at varying time periods, and therefore, a larger ratio $(\frac{\phi }{\delta })$ will correspond to a faster reacting efflux pump.

Notably, all strains that contain active AcrAB have similar efflux profiles (Fig. 6b–d). The amount of efflux in these strains is also much larger compared to the other strains. This shows the clear dominance of the AcrAB efflux pump over the other RND systems and without this pump present a strain would be much more susceptible to an antibiotic. In Fig. 7, we plot individual efflux profiles for the various pumps, combining MdsAB and MdtAB due to the minimal efflux shown from these pumps. We note that experimentally, the expression of *acrEF* has been shown to become prevalent when there is less production of AcrAB (Wang-Kan et al. 2017). Therefore to achieve these efflux profiles, we have assumed that all efflux profiles are additive. However, for the strains where AcrAB is active, we assume that AcrEF is in a down regulated state, setting $X_E=0$ (i.e. *acrEF* is not expressed). We therefore can estimate the individual efflux profiles as:

21 $$\begin{aligned} X_A=X_{AST}-X_{ST}, \quad X_E=X_{EST}-X_{ST}. \end{aligned}$$

From these efflux profiles, we can see that the behaviour for the two main efflux pumps (AcrAB and AcrEF) are very similar, showing an early increase before rapidly decreasing to reach a steady state. Interestingly, these dynamics follow closely the expression profiles of the mathematical model of the GRN in Youlden et al. (2021). Since we know that the GRN determines efflux timing and level, this strongly supports the qualitative dynamics of the model in Youlden et al. (2021). We now proceed to combine the two models.

## Multiscale Model

By creating a multiscale model of the intracellular GRN that regulates efflux with the PDE model encapsulating the substrate distribution, we can draw hypotheses on manipulating aspects of the GRN and the resulting effect upon substrate concentration in a culture (with a view to the development of novel therapeutics). By creating this multiscale model, we have a more realistic and complete model that should capture a larger range of aspects for the population’s behaviour than the previous models.

**Fig. 8:**
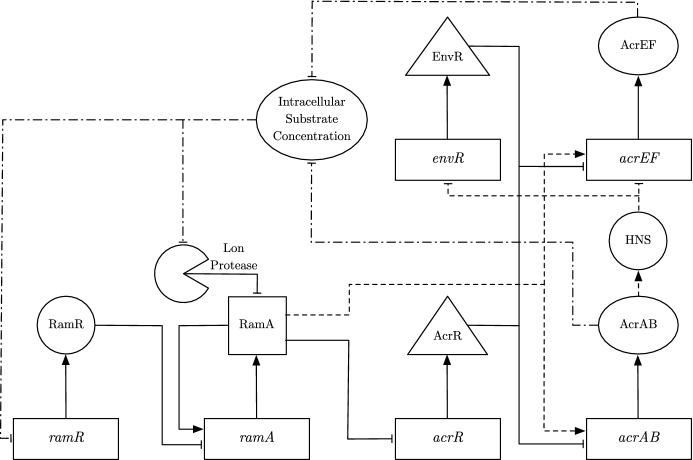
A schematic of the multiscale model. The solid lines represent mechanisms incorporated in the model of Youlden et al. (2021). The dashed lines illustrate additional mechanisms incorporated to the GRN in this study, whilst the dot-dashed lines capture the interactions between the GRN and the intracellular substrate

### Model Formulation

We plot a schematic of the updated GRN model that has substrate concentration as a signal in Fig. 8. Following our asymptotic analysis in the previous study, we have identified the genes that dominate processes within the network. Thus for simplicity, in this section we use this analysis to create a simplified version of the GRN. The adaptations we take to simplify the model are:

- Removal of the secondary transcription activators *soxS*, *marA* and *rob*.
- Removal of the post-transcriptional activator CsrA.

Our asymptotic analysis concluded that once *ramA* is expressed, these secondary transcription activators did not produce a noticeable effect at long-time on the expression of the efflux pump genes. In addition, as CsrA is not linked to other mechanisms within the network, mathematically we can easily incorporate the effect of this protein by altering the translation rate of *acrAB* mRNA. We also incorporate additional detail into the network (Dijun et al. 2018):

- RamA activates *acrEF* as well as *acrAB*.
- The link between the expression of *acrAB* and *acrEF*, is governed by heat-stable nucleoid-structuring protein (H-NS).

In regards to the first change, in the previous model the expression of *acrEF* was only governed by EnvR. Thus, we should see new interesting dynamics to the network with *acrEF* now also being dependent on the activator protein RamA to be transcribed. In regards to the second change, we have replaced our theoretical link between both efflux pumps in our previous GRN model with the molecule H-NS. When H-NS is active, it inhibits expression of both *envR* and *acrEF* (Blair et al. 2014). H-NS is believed to be involved in the experimentally observed switching dynamics of the two efflux pumps, such that *acrEF* expression is activated when *acrAB* expression is low. We include these switching dynamics by assuming that a high concentration of AcrAB will activate the molecule H-NS, hence inhibiting the expression of both *acrEF* and *envR*. To include this into our model, we assume that the variable H-NS will vary between 1 (when active) and 0 (when inactive). We represent the variable for H-NS through the following equation

22 $$\begin{aligned} \frac{\mathrm{d}H}{\mathrm{d}t}=k_h B(1-H)-m_h H, \end{aligned}$$

where $k_h$ is the activation rate of H-NS linked to AcrAB concentration and $m_h$ is the deactivation rate of H-NS. For simplicity, we take (22) to be in quasi-steady state, with $K_H=\dfrac{m_h}{k_h}$:

23 $$\begin{aligned} H=\frac{B}{B+K_H}. \end{aligned}$$

From our parameter fitting exercises, we achieved the most accurate results when efflux was dependent upon the substrate concentration, as presented in this study (see Youlden (2021) for alternative scenarios). To combine the two models, rather than choosing the substrate concentration to have a direct effect upon the expression of both efflux pumps, we choose to target the areas in the network that are deemed most likely to be affected by an antibiotic stressor:

- The internal substrate concentration inhibits expression of *ramR* (Holden and Webber 2020).
- The internal substrate concentration inhibits the concentration of Lon Protease within the cell (Holden and Webber 2020).

In regards to the first change, *ramR* is the local repressor of *ramA*, the primary activator of both efflux pumps. By targeting *ramR*, we should see an indirect effect upon *ramA* expression. For the second change, by targeting Lon Protease concentration, we indirectly affect RamA concentration by altering the protein’s degradation. In regards to the efflux of the substrate, we assume that efflux will correspond with both translation of AcrAB and AcrEF. We incorporate this by assuming that the efflux volume flow coefficient (*X*) is proportional to the combined concentrations of these efflux pump proteins. In addition, since both MdsAB and MdtAB (which were both involved in the experiments) and their corresponding genes are not captured in this GRN, we include their corresponding efflux rate from the parameter fitted equation from the cell efflux model. The equations for the combined GRN model are as follows:

24 $$\begin{aligned} \frac{\mathrm{d}R_m}{\mathrm{d}t}&= k_1\frac{K_I}{K_I+I}-\delta _m R_m, \end{aligned}$$

25 $$\begin{aligned} \frac{\mathrm{d}A_m}{\mathrm{d}t}&= k_2 \frac{K_R A+K_RK_{A_1}}{(A+K_{A_1})(R+K_R)} -\delta _m A_m, \end{aligned}$$

26 $$\begin{aligned} \frac{\mathrm{d}C_m}{\mathrm{d}t}&=k_3 \frac{K_{A_2}}{A+K_{A_2}}-\delta _m C_m, \end{aligned}$$

27 $$\begin{aligned} \frac{\mathrm{d}B_m}{\mathrm{d}t}&=k_4 \frac{K_{E_2} K_C A}{(K_C E+K_{E_2}K_C+K_{E_2}C)(K_{A_1}+A)} -\delta _m B_m, \end{aligned}$$

28 $$\begin{aligned} \frac{\mathrm{d}E_m}{\mathrm{d}t}&=k_5 \frac{K_{H}}{K_{H}+B}-\delta _m E_m, \end{aligned}$$

29 $$\begin{aligned} \frac{\mathrm{d}F_m}{\mathrm{d}t}&=k_6 \frac{K_H K_{E_1} A}{(K_H+B)(K_{E_1}+E)(K_{A_3}+A)} -\delta _m F_m, \end{aligned}$$

30 $$\begin{aligned} \frac{\mathrm{d}R}{\mathrm{d}t}&= \mu (m_1 R_m - \delta _p R), \end{aligned}$$

31 $$\begin{aligned} \frac{\mathrm{d}A}{\mathrm{d}t}&= m_2 A_m-\delta _p A -d_1 \frac{K_I}{K_I+I} A, \end{aligned}$$

32 $$\begin{aligned} \frac{\mathrm{d}C}{\mathrm{d}t}&=m_3 C_m-\delta _p C, \end{aligned}$$

33 $$\begin{aligned} \frac{\mathrm{d}B}{\mathrm{d}t}&=m_4 B_m-\delta _p B, \end{aligned}$$

34 $$\begin{aligned} \frac{\mathrm{d}E}{\mathrm{d}t}&=m_5 E_m-\delta _p E, \end{aligned}$$

35 $$\begin{aligned} \frac{\mathrm{d}F}{\mathrm{d}t}&=m_6 F_m-\delta _p F, \end{aligned}$$

36 $$\begin{aligned} \frac{{dX_{ST}}}{dt}&=\phi _{ST} \, c_B-\delta _{ST} X_{ST}, \end{aligned}$$

37 $$\begin{aligned} \frac{\partial c_B}{\partial t}&=D_B \nabla ^2 c_B-\alpha c_B+\beta c_I, \end{aligned}$$

38 $$\begin{aligned} \frac{\partial c_I}{\partial t}&=D_I \nabla ^2 c_I+\alpha c_B-\beta c_I, \end{aligned}$$

39 $$\begin{aligned} \frac{\partial c_E}{\partial t}&=D_E \nabla ^2 c_E, \end{aligned}$$

40 $$\begin{aligned} I(t)&=\frac{3}{R_M^3}\int _{0}^{R_M} r^2 \,c_B(r,t) \,\mathrm{d}r. \end{aligned}$$

Here, *I* denotes the averaged internal concentration of bound substrate. As the mRNAs and proteins in the GRN only exist in the intracellular space, for simplicity of reducing the number of unknown parameters we have opted to not include spatial effects on these variables. In regards to our GRN equations, firstly by using insights into the asymptotic analysis we have adapted Eqs. (27) and (33). From the former, we have removed activation from SoxS (also removing *soxS* mRNA and SoxS entirely from the model) whilst the latter we have removed CsrA from the translation terms of *acrAB*. We have also updated Eqs. (28) and (29), including the assumption that H-NS must not be active for mRNA transcription in both equations, whilst including dependence on RamA concentration on the latter. In addition, we have modified our basal *ramA* transcription rate in (25). Rather than including a standard basal transcription rate regardless of whether a protein is bound to the promoter region of *ramA*, we have chosen to only include transcription in the times where RamA or no proteins are bound to the promoter region. Finally, we have included the GRN influence from internal bound substrate by regulating *ramR* mRNA transcription in (24) and regulating Lon Protease degradation in (31). Whilst MdsAB and MdtAB do not feature within the GRN we include their efflux in (36), this is in order for us to replicate the full dynamics of the PDE model. We denote the variables and parameters used in this model in Tables 3 and 4 respectively. The boundary conditions used in the above model are

41 $$\begin{aligned} \left. D_B\frac{\partial c_B}{\partial r}\right| _{r=0}&=0, \quad \left. D_I\frac{\partial c_I}{\partial r}\right| _{r=0}=0, \end{aligned}$$

42 $$\begin{aligned} \left. D_I\frac{\partial c_I}{\partial r}\right| _{r=R_M}&=\left. D_E\frac{\partial c_E}{\partial r}\right| _{r=R_M}\nonumber \\&=\left( P+\frac{B+F}{X_C}+X_{ST} \right) c_I(R_M,t)-Pc_E(R_M,t), \end{aligned}$$

43 $$\begin{aligned} \left. D_B\frac{\partial c_B}{\partial r}\right| _{r=R_M}&=0, \quad \left. D_E\frac{\partial c_E}{\partial r}\right| _{r=R_B}=0. \end{aligned}$$

**Table 3:** Variables used in our multiscale model along with their respective units

Variables	Description	Units
$R_m$	Concentration of *ramR* mRNA	nM
*R*	Concentration of RamR	nM
$A_m$	Concentration of *ramA* mRNA	nM
*A*	Concentration of RamA	nM
$C_m$	Concentration of *acrR* mRNA	nM
*C*	Concentration of AcrR	nM
$B_m$	Concentration of *acrAB* mRNA	nM
*B*	Concentration of AcrAB	nM
$E_m$	Concentration of *envR* mRNA	nM
*E*	Concentration of EnvR	nM
$F_m$	Concentration of *acrEF* mRNA	nM
*F*	Concentration of AcrEF	nM
*X*	Combined efflux rate of all pumps	$\upmu$m min$^{-1}$
$X_{ST}$	Efflux rate of MdsAB and MdtAB	$\upmu$m min$^{-1}$
$c_B$	Relative concentration of bound substrate	N/A
$c_I$	Relative concentration of unbound intracellular substrate	N/A
$c_E$	Relative concentration of unbound extracellular substrate	N/A
*I*	Averaged concentration of bound substrate	N/A

**Table 4:** Parameters used in our multiscale model with their estimated values and units

Parameter	Description	Estimate	Units
$k_1$	Transcription rate of *ramR* mRNA	10	nM min$^{-1}$
$m_1$	Translation rate of RamR	1	min$^{-1}$
$k_2$	Transcription rate of *ramA* mRNA	10	nM min$^{-1}$
$m_2$	Translation rate of RamA	1	min$^{-1}$
$k_3$	Transcription rate of *acrR* mRNA	10	nM min$^{-1}$
$m_3$	Translation rate of AcrR	1	min$^{-1}$
$k_4$	Transcription rate of *acrAB* mRNA	10	nM min$^{-1}$
$m_4$	Translation rate of AcrAB	1	min$^{-1}$
$k_{5}$	Transcription rate of *envR* mRNA	10	nM min$^{-1}$
$m_{5}$	Translation rate of EnvR	1	min$^{-1}$
$k_{6}$	Transcription rate of *acrEF* mRNA	10	nM min$^{-1}$
$m_{6}$	Translation rate of AcrEF	1	min$^{-1}$
$\delta _m$	Degradation rate of mRNA	1	min$^{-1}$
$\delta _p$	Degradation rate of proteins	0.05	min$^{-1}$
$d_1$	Degradation caused by Lon Protease	0.37	min$^{-1}$
$K_R$	Dissociation constant of RamR	6.58	nM
$K_{A_1}$	Dissociation constant of RamA with *ramA* and *acrAB*	2	nM
$K_{A_2}$	Dissociation constant of RamA with *acrR*	2	nM
$K_{A_3}$	Dissociation constant of RamA with *acrEF*	60	nM
$K_C$	Dissociation constant of AcrR	20.2	nM
$K_{E_1}$	Dissociation constant of EnvR with *acrEF*	20.2	nM
$K_{E_2}$	Dissociation constant of EnvR with *acrAB*	20.2	nM
$K_{I}$	Saturation constant of Substrate	0.3	nM
$K_{H}$	Dissociation constant of H-NS	1	nM
*m*	Mutation coefficient	0 or 1	N/A
*P*	Permeability mass transfer coefficient	0.01	$\upmu$m min$^{-1}$
$X_{C}$	Efflux conversion constant	500	nM min $\mu$m$^{-1}$
$D_B$	Diffusion coefficient of bound substrate	0	$\upmu$m$^2$ min$^{-1}$
$D_I$	Diffusion coefficient of unbound intracellular substrate	2.30	$\upmu$m$^2$ min$^{-1}$
$D_E$	Diffusion coefficient of unbound extracellular substrate	1.01	$\upmu$m$^2$ min$^{-1}$
$R_M$	Membrane radius	2	$\upmu$m
$R_B$	Outer boundary radius	4	$\upmu$m
$R_M$	Membrane radius	2	$\upmu$m
$R_B$	Outer boundary radius	4	$\upmu$m
$\alpha$	Unbinding rate of bound substrate	0.22	min$^{-1}$
$\beta$	Binding rate of unbound substrate	0.09	min$^{-1}$
$\phi _{ST}$	Efflux pump formation rate for MdsAB and MdtAB	$1.57\times 10^{-4}$	$\upmu$m min$^{-2}$
$\delta _{ST}$	Efflux pump degradation rate for MdsAB and MdtAB	$2.94\times 10^{-2}$	min$^{-1}$

The first set of boundary conditions (41) represents axisymmetry in both angular dimensions for bound and unbound substrate. The boundary condition (42) represents our membrane boundary condition for unbound substrate. In this boundary condition, we have set the volume flow from efflux as $X=\dfrac{B+F}{X_C}+X_{ST}$. We have included the efflux of MdsAB and MdtAB directly as $X_{ST}$ in this equation; however for AcrAB (*B*) and AcrEF (*F*), we link their concentrations to their efflux rates by assuming a directly proportional relationship, dividing both concentrations by an efflux rate constant $X_C$. The final set of boundary conditions (43) represents no flux boundary conditions, for bound substrate at the membrane and unbound substrate at the outer boundary. The initial conditions for each concentration are as follows

44 $$\begin{aligned} c_B(r,0)&={\left\{ \begin{array}{ll} C_{B0}, &{} 0\le r\le R_M, \\ 0, &{} R_M<r \le R_B, \end{array}\right. } \nonumber \\ c_I(r,0)&={\left\{ \begin{array}{ll} \gamma \, C_{B0}, &{} 0\le r\le R_M, \\ 0, &{} R_M<r \le R_B, \end{array}\right. } \quad c_E(r,0)=0, \end{aligned}$$

where $C_{B0}$ is the initial concentration of bound substrate. We express the initial condition for the unbound substrate as a ratio of the bound substrate using $\gamma$. The initial condition for the efflux rate of MdsAB and MdtAB follows from the fitted initial condition in the previous chapter. In regards to the initial conditions for the GRN, we have down-regulated initial conditions for the mRNAs and proteins of all repressor genes (*ramR*, *acrR* and *envR*). For the mRNAs and proteins of the remaining genes (*ramA*, *acrAB* and *acrEF*), we will estimate their initial conditions in order to replicate the experiments. In the experiments, the cell has had a short amount of time to react to the substrate before the first measurement is taken, and thus, we should expect some activation of expression of the efflux genes and their activator genes. This behaviour is also shown in the parameter fitted efflux dynamics, as the initial conditions for the efflux rates of individual pumps are not fully down regulated. The notation for the initial conditions is then as follows:

45 $$\begin{aligned}&R_m(0)=C_m(0)=E_m(0)=G_{m0}, \quad R(0)=C(0)=E(0)=G_{0}, \quad X_{ST}(0)=X_{ST0}, \nonumber \\&A_m(0)=A_{m0}, \quad B_m(0)=B_{m0}, \quad F_m(0)=F_{m0}, \nonumber \\&A(0)=A_{0}, \quad B(0)=B_{0}, \quad F(0)=F_{0}. \end{aligned}$$

### Numerical Simulations

#### Parameterisation

We exhibit numerical simulations of the multiscale model, again attempting to reproduce the parameter experimental data. Since we have data for both wild-type and EST knockout strains, we compare the model against the data of these strains using parameter values from Table 4. We maintain the parameter sizes from the GRN model in the previous study (Youlden et al. 2021) as closely as possible. This is due to the extensive parameter value selection drawn from literature review and consultation with experts in the GRN (the most comprehensively studied efflux GRN at this time). In addition, we have also maintained the fitted spatial parameters from Sect. 3. We opt not to compare against the data of the remaining strains: AST (AcrAB, MdsAB and MdtAB), AET (AcrAB, AcrEF and MdtAB), AES (AcrAB, AcrEF and MdsAB), ST (MdsAB and MdtAB). These data are not used as the first three strains (which have AcrAB active) do not particularly differ in dynamics to the wild-type strain. Furthermore for the last strain, the genes that govern MdsAB and MdtAB do not feature within the GRN. To achieve the fits for the EST strain, we knock out the gene *acrAB* in the GRN by setting $k_4, m_4=0$. In addition, there is a specific strain of S. Typhimurium (SL1344) that displays MDR as a consequence of a *ramR::aph* mutation in the *ramR* gene. This strain produces a non-functional RamR protein that is unable to repress *ramA* expression, indirectly causing over-expression of the efflux pump genes. Although we do not have data for this mutant strain, we can run simulations to predict the behaviour of the strain by mutating RamR in the GRN, setting $\mu =0$. In total we simulate four strains: wild-type and an EST knockout fitted to data, and a RamR mutant. We detail the strains with their active efflux pumps and GRN mutations (if applicable) in Table 5.

**Table 5:** A summary of the strains involved in this section

Strain name	Active efflux pumps	Mutations
Wild-type	AcrAB, AcrEF, MdsAB, MdtAB	N/A
EST	AcrEF, MdsAB, MdtAB	AcrAB
RamR Mutant	AcrAB, AcrEF, MdsAB, MdtAB	RamR

We list each strain’s active efflux pumps and any mutations to their GRNs

For the following simulations, we set down-regulated initial conditions for the repressor mRNAs and proteins ($G_{m0}=G_0=0.01$nM). For the mRNAs and proteins of the remaining genes *ramA*, *acrAB* and *acrEF*, their initial conditions will vary depending on the strain we are modelling. To choose these initial conditions, we first run a simulation for each strain with down-regulated initial conditions for all mRNAs and proteins ($A_{m0}=A_0=B_{m0}=B_0=F_{m0}=F_0=0.01$nM). For the wild-type and RamR mutant strains, we expect *acrEF* expression to be down-regulated as *acrAB* is active, so we maintain the initial conditions $F_{m0}=F_0=0.01$nM. For the EST strain, the gene *acrAB* is knocked out entirely so we set $B_{m0}=B_0=0$. For the remaining variables, in the wild-type and EST strains, we choose the initial condition of the variables to be half of the maximum value over the full time course in the down-regulated simulation, i.e. the system has had a short period of time to activate from a fully down-regulated state. For the RamR mutant strain, we choose the initial condition of the variables to be the steady state values in the down-regulated simulation, i.e. with RamR mutated we always expect *ramA* and *acrAB* or *acrEF* to be up-regulated. We display these initial conditions in Table 6.

**Table 6:** Initial condition values for strains involved in this section

Strain name	$A_{m0}$ (nM)	$A_{0}$ (nM)	$B_{m0}$ (nM)	$B_{0}$ (nM)	$F_{m0}$ (nM)	$F_{0}$ (nM)	$G_{0}$ (nM)
Wild-type	3.6256	12.1458	3.1665	36.9524	0.01	0.01	0.01
EST	3.5985	12.2401	0	0	0.4798	3.5434	0.01
RamR mutant	10	25.3726	5.0964	102.0014	0.01	0.01	0.01

**Fig. 9:**
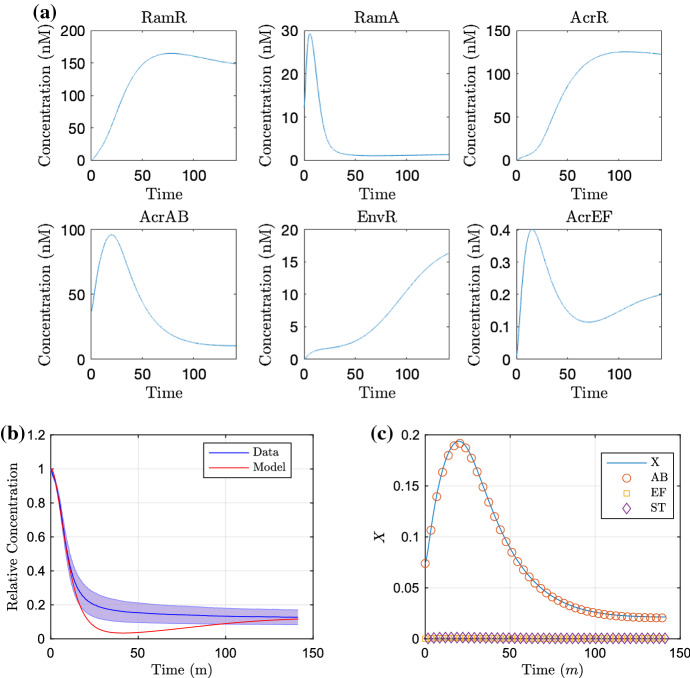
Simulations of the multiscale model for the wild-type strain, run for the time course of the wild-type data. In **a**, we show the concentration of proteins. In **b**, we exhibit the substrate concentration over time against the experimental data and **c** the corresponding efflux coefficient *X*, with the individual contributions of AcrAB (AB), AcrEF (EF) and the sum of MdsAB and MdtAB (ST) indicated (Color figure online)

**Fig. 10:**
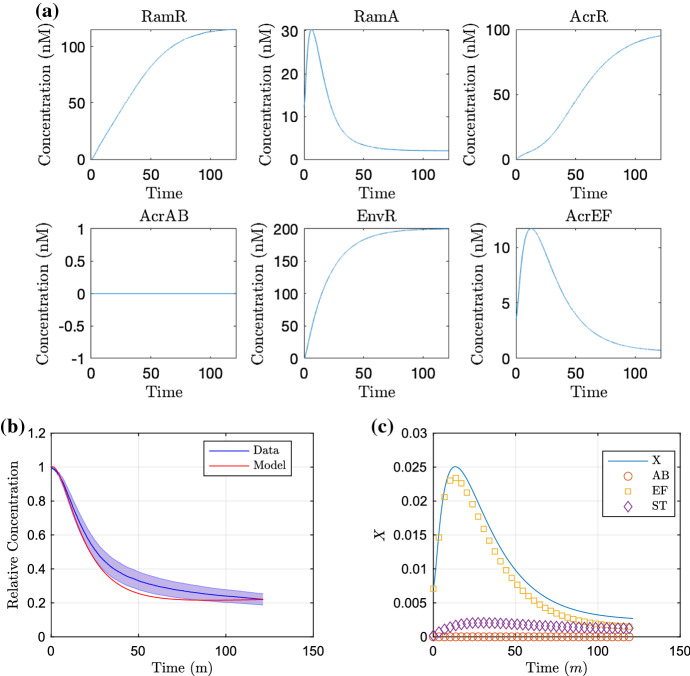
Simulations of the multiscale model for the EST strain (*acrAB* knockout, $k_4, m_4=0$), run for the time course of the EST data. In **a**, we show the concentration of proteins. In **b**, we exhibit the substrate concentration over time against the experimental data and **c** the corresponding efflux coefficient *X*, with the individual contributions of AcrAB (AB), AcrEF (EF) and the sum of MdsAB and MdtAB (ST) indicated (Color figure online)

#### Simulations

We produce simulations of the model using these initial conditions in Figs. 9 and 10 for the wild-type strain and EST strain, omitting mRNA plots (that follow closely to the protein plots) for brevity. For the wild-type strain proteins (Fig. 9a), at early time we see low concentrations of *ramR* mRNA and RamR as the gene is inactive in the presence of a high internal substrate concentration. This in turn allows fast expression of *ramA*, resulting in large concentrations of RamA protein, activating the expression of the efflux pump gene *acrAB*. Since the local efflux repressor *acrR* is inhibited by a large concentration of RamA, we see higher expression of *acrAB*. This leads to an increase in efflux rate (Fig. 9c) and resulting expulsion of substrate from within the cell (Fig. 9b). Once the intracellular bound substrate concentration is sufficiently low, we see increased activation of *ramR* expression resulting in inhibition of the expression of the network’s main activator *ramA*. The local efflux repressor *acrR* is able to express at a faster rate due to a lower concentration of RamA. The lower concentration of RamA and larger AcrR concentration result in less *acrAB* expression both indirectly and directly, respectively. This results in a decrease in efflux rate and results in an equilibrium between the transfer of intracellular and extracellular substrate. We note that the model comparison to the data (Fig. 9b) is not perfect, with mid time dynamics appearing outside the standard deviation of the data. However, we have opted not to largely differ our GRN parameter choices from the previous study by manually editing parameters as little as possible. Alternatively, we could run new parameter fitting exercises including the GRN parameters; however due to the high number of parameters within the network compared to the available data, there will likely be many non-identifiable parameters.

We show the simulation of the EST strain in Fig. 10. For (a), we note that for all time there is no expression of *acrAB* due to the gene being knocked out in this strain. Without the presence of AcrAB in this strain, there is no activation of H-NS meaning that both *envR* and *acrEF* can be expressed freely. Thus at early time, similar to the wild-type strain, with low concentrations of RamR due to a high internal substrate concentration, we see activation of *ramA* expression, with RamA this time activating expression of *acrEF*. Due to the difference in the dissociation constants of RamA with *acrEF* and *acrAB*, we see less *acrEF* expression compared to the expression of *acrAB* in the wild-type strain (Fig. 9a). The expression of *acrEF* is also reduced by the constitutive expression of *envR*, and hence, we see a lower efflux rate (Fig. 10c) than in the wild-type strain (Fig. 9c). Thus, substrate is expelled from the intracellular space at a slower rate (Fig. 10b). There is also less expression of *ramR* due to a higher intracellular substrate concentration, which reaches an equilibrium steady state with the extracellular substrate at a higher concentration than that of the wild-type strain. We note that our default parameters achieve a good comparison against the data in this case (Fig. 10b), with almost all of the model fitting within the standard deviation of the data.

**Fig. 11:**
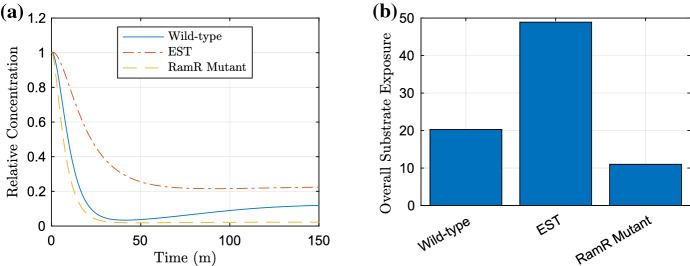
Our multiscale model showing the intracellular bound substrate concentration over time for all strains. In **a** we show time-dependent plots of all strains, in **b** we approximate the AUC of the strains using the trapezium rule, to show the overall relative substrate exposure. The wild-type strain is simulated using all parameters values in Table 4, the EST case has $k_4, m_4=0$ and RamR mutant $\mu =0$ (Color figure online)

In Fig. 11, we also produce simulations of potential RamR mutant strains and compare the resulting substrate expulsions. In (a), we plot all time-dependent simulations of the strains on one plot. In (b), we use the trapezium rule (function ‘trapz’ in MATLAB) to approximate the area under the curve (AUC) of each strain simulated in (a). The AUC shows us the overall relative substrate exposure over the simulated time course (Urso et al. 2002). Immediately we can clearly see the benefits of the RamR mutation for both strains as there is a clear reduction in substrate over all time for strains with this mutation over their counterparts (Fig. 11b). Initially, the wild-type strain and RamR mutant display similar levels of efflux (albeit the RamR mutant exhibiting slightly faster expulsion). However in the long term, the RamR mutant maintains high levels of efflux, almost eliminating all substrate from the intracellular space. This clearly shows the advantages of the RamR mutation, the strain is able to prevent large concentrations of intracellular substrate, and this is a huge contributor to its ability to exhibit MDR.

### Parameter Sensitivity

We conduct a sensitivity analysis of the parameters in our model. Here, our equation for the relative sensitivity is

46 $$\begin{aligned} S=\frac{\delta {\bar{I}}}{\delta P}, \end{aligned}$$

where $\delta {\bar{I}}$ represents the change in the steady-state value of the bound intracellular substrate concentration for the simulated strain, and $\delta P$ represents the change of the parameter being varied. If we define $P^*$ to be the default parameter value for the current parameter being varied, we then vary the parameter in the space $[0, 10P^*]$. By using a Latin hypercube method of sampling, we choose 10,000 points in the parameter space and apply these to each individual parameter, finding the relative sensitivity for each point. We choose to omit the parameters primarily involved in the spatial distribution of substrate, only varying parameters involved in the GRN, since this is the mechanism we wish to target. We also choose to omit the degradation parameters, since we have taken them to be universal to most genes in the model. Hence, varying these parameters would involve targeting all genes simultaneously which is not only an unrealistic target but will exhibit similar behaviour to preventing the whole network from being expressed.

**Fig. 12:**
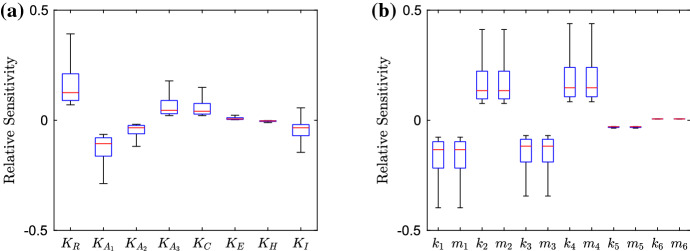
Box plots showing the relative sensitivity of parameters involved in the GRN for the wild-type strain, varying parameters in the region $[0, 10P^*]$, where $P^*$ is the default parameter value. In **a**, we depict the dissociation and saturation constants; in **b**, we depict the various transcription and translation rates related to mRNAs and proteins (Color figure online)

#### Wild-Type Strain

We exhibit the parameter sensitivity results using the steady state for the wild-type strain with box plots in Fig. 12. In (a), we denote the dissociation and saturation constants involved in the model, we can immediately see that the dissociation constant related to RamR ($K_R$) is the most sensitive. By varying this parameter, we should see direct effects on the activation of *ramA* transcription. It is interesting to note that the sensitivity of this parameter is larger than that of any of the dissociations constants related to RamA ($K_{A_i}$). Thus in this strain, targeting *ramA* expression via a repressor to reduce the genes expression may be a more effective method than targeting the RamA binding process to the promoter regions of various other genes in the network. However, we must note that the sensitivity of all RamA dissociation constants is still significant, with the dissociation with *ramA* and *acrAB* ($K_{A_1}$) showing more sensitivity than the dissociation with *acrR* ($K_{A_2}$) and *acrEF* ($K_{A_3}$). This is expected, as in this wild-type strain we have normal levels of *acrAB* expression, so we should expect lower expression of *acrEF* and hence a smaller sensitivity upon parameters related to the gene. We note that the other two parameters with notable significance are related to AcrR ($K_C$) and substrate ($K_I$). The first of these is expected due to direct inhibition of expression of the efflux pump gene *acrAB*. Regarding $K_I$, we do not know the full mechanisms behind how the substrate interacts with the network, but this could provide insight for potential further research. We show transcription and translation rates in (b). We note that for each individual gene the transcription rates show similar sensitivity to the translation rates. Thus when targeting gene expression, both transcription and translation seem to be feasible targets. It is clear that the wild-type system is the most sensitive to the expression of four genes, namely *ramR* and *ramA*, *acrR* and *acrAB*. This gives us clear insights into the most important genes to target when inhibiting efflux in a wild-type strain. The strain exhibits little sensitivity to *envR* and *acrEF*. This again is expected, as with no major restrictions upon *acrAB* expression we expect H-NS to be prevalent and the expression of *envR* and *acrEF* to be minimal.

**Fig. 13:**
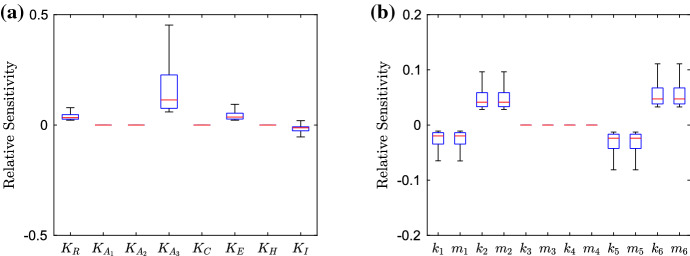
Box plots showing the relative sensitivity of parameters involved in the GRN for the EST strain, varying parameters in the region $[0, 10P^*]$, where $P^*$ is the default parameter value. In **a**, we depict the dissociation and saturation constants; in **b**, we depict the various transcription and translation rates related to mRNAs and proteins (Color figure online)

#### EST Strain

We exhibit the parameter sensitivity results using the steady state for the EST strain in Fig. 13. Due to the gene being knocked out, we can immediately see that all parameters involved with *acrAB* have lost all sensitivity. In (a) the most sensitive parameter is the dissociation of RamA with *acrEF* ($K_{A_3}$). Which is due to AcrEF being the main active efflux pump in this strain. Compared to the wild-type strain (Fig. 12), we can see decreased sensitivity to the dissociation of RamR ($K_R$). This could be due to RamA having a smaller activation effect on *acrEF* expression than *acrAB* expression, thus inhibition of *ramA* expression from RamR would have less of an effect on efflux expression. We also see increased sensitivity of the dissociation of EnvR from *acrAB* and *acrEF*. With *envR* being constitutively expressed in this case, we should expect higher sensitivity from this local repressor. In (b), we note the most sensitive parameters are related to the expression of *ramR*, *ramA*, *envR* and *acrEF*. The strain shows similar sensitivities to all of these genes and thus targeting any of their expressions should be a viable target for inhibiting efflux. Notably however, the strain is most sensitive to *envR* and *acrEF*, leading us to believe that repressing *acrEF* expression directly or via *envR* may be a more effective target than preventing activation (via RamA for example) of the gene’s expression.

**Fig. 14:**
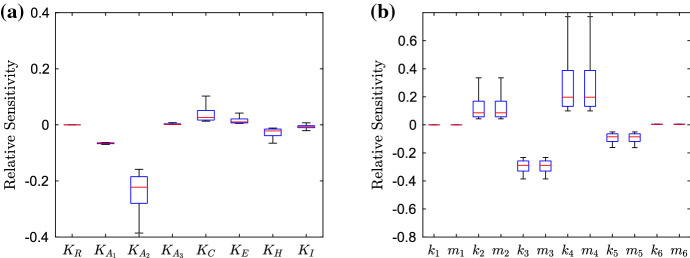
Box plots showing the relative sensitivity of parameters involved in the GRN for the RamR mutant strain, varying parameters in the region $[0, 10P^*]$, where $P^*$ is the default parameter value. In **a**, we depict the dissociation and saturation constants; in **b**, we depict the various transcription and translation rates related to mRNAs and proteins (Color figure online)

#### RamR Mutant Strain

We exhibit the parameter sensitivity results using the steady state for the RamR mutant strain in Fig. 14. Immediately, compared to the wild-type strain (Fig. 12) we can see that all of the parameters relating to *ramR* ($K_R$, $k_1$ and $m_1$) all have no sensitivity due to the mutations of RamR. Additionally, the sensitivity of the desaturation constant relating to substrate ($K_I$) is reduced, which could be due to the substrate only now having an effect on Lon Protease concentration. In (a), we can see that the dissociation of RamA with *acrR* ($K_{A_2}$) is the most sensitive, this is interesting to note as the previous study (Youlden et al. 2021) uncovered this link as one of the key mechanisms for activating efflux in this mutant strain. The other RamA dissociation parameters range of sensitivity are both decreased compared to the wild-type strain, which could be due to the higher concentration of RamA in this strain, such that large activation will occur regardless of the dissociation constant. We note the sensitivity of dissociation related to *acrEF* ($K_{A_3}$) is minimal, which is expected due to high *acrAB* expression in this strain. We also see an increase in the dissociation of H-NS ($K_H$), which could be due to a high concentration of RamA. If H-NS does not inhibit *acrEF* expression so strongly, we would see higher activation of *acrEF* expression through large concentrations of RamA. In (b), we can see that the most sensitive parameters are related to the expression of *ramA*, *acrR* and *acrAB*. The strain is most sensitive to *acrAB* expression, which we would expect as it is direct expression of one of the efflux pumps, which is over-expressed in this strain. Interestingly, there is increased sensitivity to changes in *envR* expression compared to the wild-type (Fig. 12). Whilst we have hardly any expression of *envR* in this strain, the sensitivity increase here could be due to the overexpression of *acrAB*. Such that any repression of *acrAB* will have a larger change in concentration compared to the wild type. We note that both RamR mutant and wild-type strains exhibit similar sensitivity to changes in *ramA* and *acrR* expression, even with the differences to their GRNs.

### Network Manipulation

**Fig. 15:**
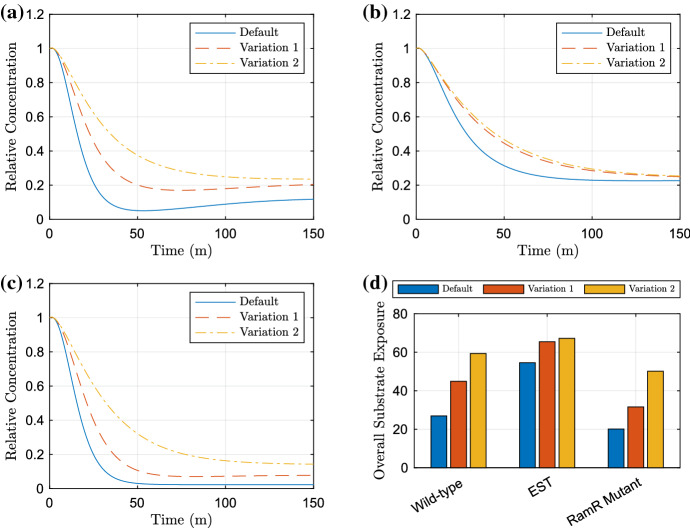
Plots exhibiting the effects of varying *ramA* and *envR* expression on the bound intracellular substrate over time. The default parameters are $k_2=10$ and $k_5=10$, variation 1 are $k_2=1$, $k_5=10$ (*ramA* expression inhibited) and variation 2 are $k_2=1$, $k_5=100$ (*ramA* expression inhibited and *envR* expression promoted). In **a**, we have the wild-type strain, **b** the EST strain, **c** the RamR mutant strain. Finally in **d**, we exhibit the AUC between the default and each of the manipulated parameter value simulations for the strains (Color figure online)

Whilst the parameter sensitivity analysis has given us insight into the sensitivity of the substrate at steady state, it is important to note that we do not know the effects caused by manipulating parameters through the rest of the time course. Therefore, we take the parameters to which the model is most sensitive in the above analysis and plot relevant time-dependent simulations. In Fig. 15, we show the effects of varying either *ramA* expression or *ramA* and *envR* expression. We have chosen these targets as they proved to be sensitive to all strains in the parameter sensitivity analysis. For a more comprehensive analysis of possible perturbations, see Youlden (2021).

We can see that these manipulations have clear effects on the dynamics in all strains, although the EST strain (Fig. 15b) does show a lesser change in dynamics to the wild-type and RamR mutant strains (Fig. 15a, c). In addition, the total substrate exposure (15d) has similar differences in exposure for one manipulation in all strains; however, multiple manipulations have larger effects to the wild-type and RamR mutant strains than the EST strain. These differences are likely due to there being one efflux pump already inactive in the latter strain, meaning less efflux inhibition is required. In the RamR mutant strain (Fig. 15c) however, we see much larger changes to the dynamics compared to the non-mutant strains (Fig. 15a, b). This is a useful insight as the RamR mutation has experimentally been shown to cause MDR. We note that in all strains there is a notable difference in the mid time dynamics, with the efflux rate of substrate slowed. Thus, in regards to an antibiotic substrate, the timing of slowing efflux may be crucial. If cells within the culture cannot expel enough antibiotic at a fast enough rate, the antibiotic may have already caused irreversible damage to the cells and hence the cells may die even if they are able to pump out enough antibiotic to a low enough concentration that would normally be under a killing threshold, thus preventing MDR in the strain.

## Discussion

Antibiotic resistance is a continually growing threat to global public health, such that the WHO are predicting that we are heading to a post-antibiotic era (World Health Organization 2015). In *Salmonella*, it has been shown that MDR is commonly conferred via the over-expression of RND efflux pumps (Blair et al. 2014). Inhibition of these efflux pumps however is not a simple process, it has been demonstrated that inhibition of one RND system results in efflux through another of the five RND systems (Hirakawa et al. 2008; Nishino 2016). It is important to understand the contribution of each of these systems towards MDR in order to create an effective treatment strategy that optimises the usage of antibiotics.

In this paper, we have considered multiple strains of *Salmonella*. These strains include a wild-type as well as various knockout strains containing combinations of RND systems. Efflux assays have been performed on these strains, using a shared substrate (ethidium bromide) of the RND systems. We have formulated a model to replicate the behaviour of the *Salmonella* strains in these experiments. Parameter fitting techniques showed the model matched the dynamics of the experimental data, encapsulating the behaviour of all *Salmonella* strains and revealing their efflux activity. Notably all strains that contained active *acrAB* displayed a similar efflux profile. The model showed that these strains also exhibited much larger peaks of efflux over the course of the experiment compared to strains with inactive *acrAB*. This agrees with AcrAB-TolC system being the most dominant RND efflux pump in *Salmonella*. The model suggests that it is upregulated to much higher levels than the other efflux pump systems and in terms of inhibiting efflux it should be the highest priority target. In regards to the other strains with inactive *acrAB*, we see a drop to very minimal efflux in the strain with *acrEF* also inactive. The model therefore suggests that the efflux pumps MdsAB and MdtAB contribute very little to substrate efflux and therefore are a low priority target for inhibiting efflux. Additionally, this suggests that when the AcrAB-TolC system is not present the AcrEF-TolC system is the most dominant RND system and therefore should be a secondary priority target for inhibiting efflux. Furthermore, we recognised that the efflux profiles were similar to simulations of the expression of genes from the GRN model of our previous study (Youlden et al. 2021). In addition, the model produced the best results when efflux volume flow was dependent on the intracellular substrate concentration. This has given us confidence that the existing GRN model is appropriate and that gene expression is driving the behaviour of efflux in these experiments.

By combining the two models of the GRN and substrate efflux, we created a multiscale model that encapsulates the behaviour of how a *Salmonella* culture expresses genes in order to react to a substrate stressor. We incorporated this through linking the intracellular substrate concentration to the expression and degradation of *ramR* and RamA, respectively. By producing this model, we were able to consider an additional strain (SL1344) that displays MDR due to a mutation in the *ramR* gene causing non-functional RamR protein. We predict this strain’s behaviour by adapting parameters to replicate the GRN of this strain. Simulations exhibited the higher potential for resistance in the RamR mutant strain. The model suggests that intracellular substrate is expelled quicker, to a lower concentration and a significant decrease in overall exposure in the RamR mutant compared to the other strains. This shows the challenges we face to combat MDR strains and the importance of targeting increased resistance dynamics when producing novel treatment strategies.

By conducting a parameter sensitivity analysis on parameters in the GRN, we were able to identify the most suitable targets for inhibiting efflux for all strains. The model showed us that the largest sensitivity common between all strains was towards the genes *ramA* (the main activator gene of *acrAB*) and *envR* (a repressor gene of *acrEF* and *acrAB*). This agrees with the conclusions of the previous study (Youlden et al. 2021), as the same genes were considered the highest priority targets in the GRN to combat antibiotic resistance. By producing time series simulations varying the expression of these genes, we were able to show the behaviour of the strains under the effects of a potential adjuvant. Interestingly, one ten-fold manipulation of *ramA* expression in the RamR mutant strain achieves a higher overall substrate exposure than the default wild-type strain with this parameter choice. Therefore, the model predicts that a single target may be sufficient to combat MDR in the RamR mutant strain. Alternatively, given that targeting *envR* and *ramA* simultaneously produced even clearer improvements in predicted substrate exposure in the wild-type and RamR mutant strains, the model predicts that a combination of adjuvants (if achievable) could have quicker and more significant results. Furthermore, the model showed that manipulations caused larger differences to the mid time dynamics of substrate expulsion. Therefore, the utilisation of timing and concentration of doses to coincide with the largest drop in expulsion could be vital to produce an effective treatment method.

In summary, we have created a spatial model that has accurately replicated the dynamics of efflux assay experiments. This model lends weight to a priority ordering of the RND efflux pump systems for inhibiting efflux in *Salmonella*. By producing a multiscale model combining the spatial model with an existing GRN model, we were able to simulate and predict the behaviour of a MDR strain. Model analysis has given us insights on how best to use a novel adjuvant to optimise antibiotic treatment. By creating this model we have provided a basis for understanding efflux-mediated MDR in *Salmonella* with multiple opportunities for further research. The model provides the ability to target or manipulate any area of the GRN or cell spatial structure to achieve new hypotheses on how the culture will react to an antibiotic. We could therefore use this model to simulate other MDR strains and gain insights on the best targets within these new strains to prevent drug resistance. Furthermore, the spatial model provides the framework to be applied to different bacteria genera which could in turn be combined with new GRN models to generate similar hypotheses. In addition, we could introduce the administration of a potential treatment strategy to the model. This could be in the form of an adjuvant targeting the previously mentioned priority genes in the GRN, or a synthetic molecule directly slowing efflux activity or altering membrane permeability. We have shown that the model in its current format does a good job of replicating *in vitro* experiments, it would be important future work to also apply the model to *in vivo* situations. This could be incorporated instead of considering drug infusion, using a time-dependent drug source and analysing the activation of efflux pumps through drug clearance. In addition, it would be instructive to consider potential differences in the parameters underpinning individual cells within a population: by selecting parameter values from relevant parameter distributions, we could consider how likely subpopulations of cells at the extremities might be to escape efflux suppression for example. We believe that this model has broadened our knowledge in efflux-mediated MDR in *Salmonella* leading to hypotheses on the production of novel treatment methods to combat antibiotic resistance. Predicting the effect of these on efflux overall is complex given the nonlinear interplay between different efflux pumps in any given strain; the model presented here provides a computational framework (that can also be readily adapted to other strains of bacteria) with which to test these.
